# The Treatment of Extra-Uterine Pregnancy

**Published:** 1917-04

**Authors:** Aime Paul Heineck

**Affiliations:** Chicago, Ill., Professor of Surgery, Chicago College of Medicine and Surgery; Surgeon to Douglass, Jefferson Park and Francis Williard Hospitals


### The Treatment of Extra-Uterine Pregnancy.

BY AIME PAUL HEINECK, M. D., CHICAGO, ILL., PROFESSOR OF SURGERY, CHICAGO COLLEGE OF MEDICINE AND SURGERY; SURGEON TO DOUGLASS, JEFFERSON PARK AND FRANCIS WILLIARD HOSPITALS.

Extra-uterine pregnancy is a condition which obtains in the female when a fertilized ovum is permanently arrested somewhere in its course from the ovary to the uterus and undergoes development at this point of arrest or abnormal lodging place.

This condition occurs far more frequently than is generally believed. It is often overlooked, often misdiagnosed. Inflammation and sepsis following a supposed miscarriage are often due to a ruptured extra-uterine pregnancy.

So continuously destructive is the action of the syncitial cells upon the tubal wall that tubal pregnancy is compared by many clinicians to a parasitic growth, to a malignant process, requiring in every case to be treated as such.

Extra-uterine pregnancy almost invariably is primarily tubal; ovarian pregnancies occur, but they are pathological rarities. Tubal pregnancy occurs in all races and at all periods of child-bearing age. It may be intramural, isthmic or ampullary; unilateral or bilateral. It does not seem to show any predilection for either tube. It may be the individual's first conception, or precede, be associated with, or follow one or more normal uterine pregnancies. Normal uterine pregnancies have intervened between two extra-uterine pregnancies. Some cases have been preceded by one or more miscarriages, accidental or induced; may have occurred in primiparae and multiparae, after a prolonged period of sterility, and in a considerable number of cases one obtains history of previous inflammation, gonorrheal or other, of the internal genitalia. It is thought that inflammatory processes act as etiological factors by destroying the tubal cilia and by producing kinks, strictures, adhesions, and obliterations of the tube.

Every conceivable variation has been observed in cases of tubal pregnancy; it has been repeated in the opposite tube, in a tubal stump. There has been simultaneous gestation in the right and left tubes and also simultaneous tubal and normal uterine pregnancies. Under the designation, "Tubal Twin Pregnancy," three distinct conditions are included:

1. Where one ovum is intra-uterine and the other extra-uterine.

2. Where each tube contains an ovum.

3. Where both ova are contained in one tube.

Tubal pregnancy at times simulates, and in some of the reported cases was associated with one or more of the following pathological conditions: ovarian cyst, salpingitis, pyosalphinx and hydrosalphinx of the opposite side, appendicitis, various forms of uterine displacement, and various uterine neoplasms.

What are the possible terminations of an extra-uterine pregnancy abandoned to the unassisted resources of nature?

1. The pregnancy may go to term and a living child be delivered through channels created by the surgeon. In connection with this termination, one must keep in mind that extra-uterine children frequently die in the first few days of life; many of them have lived only for a few hours. They are frequently the subject of deformity. The operation necessitating their removal from the maternal organism may prove fatal to them. It may prove fatal to the mother either immediately from the surgical shock or from excessive hemorrhage; or remotely from toxemia, septicemia or pyemia.

2. The pregnancy may go to term and the child remaining undelivered dies and persists indefinitely in the maternal organism. It may at any time become a menace to the life and health of the mother.

3. The fetus may die previous to term. Small embryos when expelled into the peritoneal cavity are promptly absorbed, unless the placenta retains a firm attachment to the tube or contracts new attachments. Fetuses that die at an advanced state of development cannot be absorbed.

The undelivered tubal fetus may undergo—

(a) Putrefaction.

(b) Mummification.

(c) Maceration.

(d) Transformation into lithopedion.

(e) Septic changes:

1. From communication with neighboring organs.

2. From contiguity with neighboring organs.

After the death of the fetus, the liquor amnii is absorbed. No more is secreted. The cyst shrinks and the gestation sac may be considered as a cyst—a fetal cyst. The fetal cyst may be merely:

1. A mechanical inconvenience to the maternal organism.

2. An obstacle to a subsequent intra-uterine pregnancy, and have to be removed to allow a simultaneous or subsequent uterine pregnancy to go to term.

3. A source of irritation to one or more contiguous organs, causing rectal or vesical disturbances, by compressing the intestines, determining an ileus; by compressing the bladder or ureters, causing urinary retention.

4. A cause of uterine displacement.

5. A cause of diagnostic errors.

6. The fetal cyst walls may and frequently do become adherent to surrounding organs and tissues, and the cyst by means of a perforative inflammation opens into the (a) bladder; (b) vagina; (c) intestinal canal, or (d) through the abdominal wall by either or by several of which channels of outlet it eventually completely or incompletely eliminates its decomposing contents.

Rupture is one of the terminations of tubal pregnancy. It is appalling in its suddenness and often overwhelming in its results. It may occur before or after the death of the fetus. Primary or secondary gestation sacs may rupture. The gestation may be arrested by this accident or may continue uninterrupted though changed in anatomical type. Rupture is associated with hemorrhage, circumscribed or diffuse, belonging to one of the three following types, or to a combination of two or of all these types: (a) Extra-tubal; (b) intra-tubal; (c) intra-mural. If the amniotic sac be ruptured and there be an outflow of the amniotic fluid, gestation will come to an end.

Extra-tubal rupture takes place:

(a) Into the peritoneal cavity: if the ovum does not perish, the pregnancy is continued as a tubo-peritoneal or peritoneal pregnancy.

(b) Between the folds of the broad ligament. If the gestation be not interrupted, it will continue as an intra-ligamentary or tubo-abdominal pregnancy. Intra-ligamentary pregnancy is far more infrequent than peritoneal pregnancy. The layers of the broad ligament provide a ready-made capsule by means of which the amount of bleeding is restricted.

(c) An intra-mural rupture may lead secondary to a intra-tubal or extra-tubal rupture. In intra-mural rupture, a thin layer of muscle tissue and the peritoneum separate the blood sac from the peritoneal cavity, or from the intra-ligamentary space. The condition is somewhat analogous to that which obtains when a saccular aneurysm ruptures and the blood escapes interstitially. If the abdominal opening of the tube be occluded, intra-tubal rupture leads to an accumulation of blood in the cavity of the tube, viz.: hematosalphinx. If the abdominal end of the tube be not occluded, the blood passes out of the tube into the peritoneal cavity, giving us a pelvic hematocele or a hemoperitoneum. The ovum, continuing to develop in the tube, may rupture secondarily either into the peritoneal cavity or between the folds of the broad ligament, and gestation therein continue. If one variety of rupture fails to relieve the tension, the gestation sac ruptures in another direction. In tubal abortion the ovum is carried out of the tube by the intra-tubal hemorrhage.

Tubal ruptures and tubal abortions are associated with hemorrhage. Hemorrhage may also result from the perforation of the tubal wall by the development of the chorionic villi. The amount of blood discharged bears no relation to the extent of the rupture. Severe and even fatal hemorrhages have occurred from very small orifices. The rupture may be punctiform in size; may be a large tear; may be almost a complete rending of the tube. Rupture in the peritoneal cavity leads either to the formation of an hematocele, or to a flooding of the peritoneal cavity; the latter will prove fatal if the hemorrhage be not operatively arrested. Maternal death may be caused by one severe intraperitoneal hemorrhage, or by recurring hemorrhages. The signs and symptoms of acute anemia are quickly produced.

If the extra-tubal rupture be between the folds of the broad ligament, a pelvic hematoma will result. This hematoma is almost invariably one-sided and, needless to say, is on the side of the rupture. In some cases, it dissects forward between the uterus and the bladder, or backward around the uterus beneath the peritoneum, and extends to the opposite side. If tension within the hematoma is excessive, rupture may take place secondarily into the peritoneal cavity, giving us the combined condition of both intra-peritoneal and extra-peritoneal hemorrhages. The same may occur in a hematosalpinx. Excessive tension leads to tubal rupture either into the peritoneal cavity, or between the folds of the broad ligament, or in both directions. These encysted blood collections—hematomata or hematoceles—are partially or completely absorbed, persist as fibrous bands or masses, or become infected and lead to pus formation. If the suppurative inflammation be circumscribed, an abscess is formed. Should the inflammation spread to, or the abscess burst into the peritoneal cavity, there results a circumscribed or diffuse suppurative peritonitis. Should the inflammation extend to the retro-peritoneal connective tissue, a cellulitis results, with all its accompanying dangers.

The migration of the ovum into the abdominal cavity, through the ostium abdominale, is known as tubal abortion (Bland Sutton). Tubal abortion may also lead to hematosalpinx. Usually, however, the blood escapes freely through the ostium abdominale into the cul-de-sac of Douglas, and either becomes encysted there, or escapes into the general peritoneal cavity. The three factors involved in causing abortion or rupture apart from such extraneous causes as bimanual examination, etc., are:

1. The destructive action of the trophoblasts.
2. Bleeding.
3. Contraction of the muscular wall of the tube.

One should always be very careful and gentle in examining a case of probable tubal pregnancy; the danger of rupturing one of these tubes by rough manipulation exists.

Tubal abortion may be complete or incomplete. In the former, there is usually one attack of pain and weakness. In the incomplete form, we have repeated attacks of weakness. The abortion, if the amniotic sac remains intact, and if the ovum resists absorption, leads to a tubo-peritoneal or a peritoneal pregnancy. If the villi or placental attachments are destroyed, the ovum being unable to form secondary attachments to other structures, dies.

TREATMENT.

Extra-uterine pregnancy is as truly a surgical disease as appendicitis, and though, as in this disease, clinical cure may at times be obtained by non-operative measures, it is not common for that clinical to be an anatomical cure. In ectopic pregnancy, do not consider the viability of the child except as it endangers the life of the mother. We must destroy the fetus to save the mother. Without surgical aid, extra-uterine pregnancy always terminates fatally to the child, and frequently causes the mother's death.

Nature's tedious methods of relief, and the many dangers to which the woman is obviously exposed during its occurrence, justify surgical interference. Even the absorption of large uninfected collections of blood is far more prolonged than postoperative convalescence. The ideal time for operating is before the rupture takes place. Error should be made upon the side of prompt operation rather than on that of undue waiting.

In the hands of the average operator, the only possible dangers to which the mother is exposed by the operative removal of the dead or alive ectopic fetus are sepsis, hemorrhage and shock. The first can be avoided, the second can be completely controlled, and the third can be minimized and almost always overcome.

Some operators make use of the terms "primary laparotomy" and "secondary laparotomy." In the former, the operation is performed during the life of the fetus. It is in accord with the theory and practice of modern surgery. It attacks tissues while they are healthy, in preference to awaiting nature's blind efforts to improve conditions. Secondary laparotomy is the operation performed after the death of the fetus.

The diagnosis of ectopic gestation is in itself an imperative indication for operation. Delay is inadmissible. The longer one waits, the more dangerous the condition becomes. A waiting policy is often fatal. Lives can be saved by accurate diagnosis, prompt decision, and skillful operating. The profession in general has not exhibited that keenness and alertness toward extra-uterine pregnancy which has characterized its study of appendicitis in the last few years.

In tubal ruptures and in tubal abortions, the first indication is to stop the hemorrhage. This indication is urgent. Hypodermic medication will not accomplish it. To stop this hemorrhage, place not your faith upon the coagulability of the blood, the lessened force of cardiac action, or such agents as heat, cold, styptics, and the like. Stimulants must not be used in internal hemorrhage until the bleeding vessels have been secured, as increase of cardiac action and of arterial tension would be followed by recurrence of bleeding. Open the abdomen; stop the hemorrhage by ligating bleeding points.

Even in the absence of urgent symptoms, do not delay operation. To postpone the operation is to incur adhesions and hematocele sacs in their various forms. As long as the embryo or fetus lives, the placenta increases daily in size, in vascularity, and in difficulty of removal. Furthermore, the increasing size of the child and of the placenta adds to the danger of secondary rupture.

Having decided to operate, two pathways are open: 1. Through the vaginal wall. 2. Through the abdominal wall. In some difficult cases, you may have to use both the abdominal and vaginal route. We recommend the vaginal route in the following conditions, viz.: pelvic abscess—when the gestation sac has been converted into a pelvic abscess, when suppuration has occurred in an intra-ligamentary fetal cyst, and in all intra-ligamentary hematomata. The opening of the pelvic abscesses by way of the vagina is a safe and wise surgical procedure. The results are almost always satisfactory. 3. In those cases where the fetal parts closely press against the vaginal wall. Even here it may be necessary to make use of the abdominal route, in addition to the vaginal route. It is often impossible to remove the impregnated tube through a vaginal incision. There is always greater danger of wounding the intestines when one makes use of the vaginal route. We prefer the abdominal route because it enables the operator: 1. To remedy at the same time coexisting pathological conditions, hydrosalphinx, obliteration of the abdominal ostium of the unaffected tube, ovarian cysts, etc. 2. To more thoroughly and more rapidly arrest hemorrhage. 3. To make a more direct examination and thereby judge better the extent of damage, and formulate a more accurate diagnosis, and effect a more conservative ablation of organs. 4. To have the operative field under much better control, to more quickly come in contact with the condition, and to better and more completely remove the fetal sac and its contents. The separated ovum or liberated fetus may ascend in the abdominal cavity, and it may be very difficult to find and remove it by the vaginal route. An abdominal incision enables the operator, in case of an incorrect diagnosis, to treat these conditions that simulate ectopic gestation. In operating, sight as well as touch, is a very useful aid.

The greatest difficulty that we encounter near term, at term or after term in operating for ectopic gestation is connected with the removal of the placenta. A slight detachment of the placenta often results in alarming hemorrhage.

We make use of the supra-pubic or infra-umbilical. Avoid cutting the urachus. Avoid cutting the epigastric vessels. Cutting into a patulous urachus is as significant as cutting into a urinary bladder. The cut must be repaired. Make timely and appropriate use of the Trendelenburg position. The patient must be placed in this position gradually, not suddenly. The return to the horizontal position must also be gradual. The Trendelenburg position facilitates the gravitation of the intestines toward the diaphragm. It permits a better view of the pelvic tissues or organs. After the patient has been gradually placed in the Trendelenburg position, the intestines and general peritoneal cavity are walled off from the pelvic cavity by gauze pads.

In all operations for extra-uterine gestation, the opposite tube and ovary should be carefully examined, as they may be the seat of degenerative changes.

Never make a needless sacrifice of tissues or organs. In the absence of the positive indication, such as a highly contracted pelvis, preventing the birth of a living child through the natural channels, etc., never remove the non-diseased tube and ovary. As most extra-uterine pregnancies are tubal, early operation will permit the preservation of the ovary. The preservation of the ovaries is of a benefit to the patient.

The main difficulty in early and late operations is hemorrhage. The ideal treatment for hemorrhage incident to operations undertaken for the removal of an ectopic gestation sac is prophylaxis. Therefore, do not provoke uncontrollable hemorrhages. Proceed after having well sized up the situation. Hemorrhage must be controlled by ligation or by compressing the bleeding points. Normal salt solution must not be given, either intravenously, subcutaneously or per rectum, before the bleeding points have been controlled or secured. Once the bleeding is under control, its use is of signal benefit. It increases the volume of circulating fluid. Do not close up the abdomen until you are satisfied concerning the hemostasis. Hemorrhage is most profuse if the fetus be alive at the time of operation.

If possible, do not leave denuded peritoneal surfaces. They are possible avenues of infection. After ablation of one or both tubes, suture the folds of the broad ligament to each other from the superior pelvic strait to the angle of the uterus. Peritonization—that is, the covering with peritoneum of all denuded surfaces, lessens adhesion formation. These adhesions may be attended with colicky or other pains; may cause intestinal obstruction. This peritonization lessens the hemorrhage and creates a barrier capable of limiting the extension of inflammatory processes.

In attempting to remove the fetal sac and its contents, be careful lest these efforts inflict much damage upon contiguous organs. Repair such damage, if feasible, before closing up the abdominal cavity.

In early, unruptured, tubal pregnancy, there are usually no adhesions. If adhesions be present, they are to be separated, as in all other intra-abdominal surgical interventions, with great care and by the same methods. The incision, about three inches in length, is carried through the different layers of the abdominal wall into the peritoneal cavity. The first step is to locate the uterus. Using the fundus of the uterus as a guide, and proceeding to the right and to the left, examine both tubes and both ovaries. Separate all adhesions, if such exist, of the gestation sac to contiguous organs. Then remove the gestation sac (which is usually tubal), as a whole, if possible, by total resection of the Fallopian tube involved. Suture the folds of the broad ligament; leave no denuded peritoneal surfaces. Close up the peritoneal cavity. Post-operative treatment is that of uncomplicated laparotomy. If the pregnancy be ovarian in type and be early and unruptured, do a typical ovariotomy. Accuracy and rapidity in operating is as essential in these cases as in any other intra-abdominal work.

If the gestation sac is ruptured and hemorrhage has occurred or is occurring, after opening the abdominal cavity immediately locate and keep in view the fundus of the uterus. Determine on which side is the ruptured gestation sac. Seize the uterus, preferably with the hand or with a double tenaculum. It is a most important landmark. Having determined on which side the rupture is (it is usually tubal), apply a clamp at the uterine end of the tube. This will stop all further hemorrhage from the ovarian artery of that side. Apply another clamp immediately below the tube, compressing the folds of the broad ligament, but not injuring the ovary. Then remove the affected tube and the gestation sac. Ligate all bleeding points, suture the folds of the broad ligament and the tubal surface of the uterine stump. Remove as expeditiously as you can the easily removable liquid blood and blood clots contained in the peritoneal and pelvic cavities. Remove the embryo if it can be found without prolonged search. Let there be no needless exposure, no needless traumatizing of the intestines. Mechanical, chemical and thermal irritation of the peritoneum intensify operative shock and may be followed by the aperistaltic form of ileus. Post-operative treatment is that of acute internal hemorrhage for which laparotomy has been performed. Use normal saline solution secundum artem.

The most dangerous operative conditions, from the maternal standpoint, are presented by those cases in which the fetus is alive. In these cases the hemorrhage frequently is appalling. Some authors have suggested that the abdominal aorta be compressed. When the placenta is attached to the line of incision, the hemorrhage is profuse; it is checked by firm compression. In those cases, in which the fetus is alive, we have two things to accomplish, and they must be accomplished with the preservation of the mother's life. The first thing to accomplish is the removal of the living child. The last and most important is the removal of the ovular debris—placenta, membranes, etc. One is not often called upon to operate on cases in which the living child is present. For a physician knowingly to abstain from operating in a case of extra-uterine pregnancy before it reaches term is, to say the least, injudicious. The best practice is to terminate these pregnancies early, before the development of the ovum is much advanced.

Remove the fetus without disturbing the placenta. If the fetus is alive, after having opened the abdominal cavity and protected the peritoneal cavity by compresses from the outflow of amniotic fluid, ligate the umbilical cord as in a normal pregnancy and remove the fetus. Have the amniotic fluid escape externally as much as possible. Upon the maternal end of the umbilical cord a clamp is placed, the umbilical cord being cut either between the ligature and the clamp or between the two clamps.

If the fetus has reached term or near term and is dead, there is some difference of opinion as to which operation is preferable—the immediate operation or the delayed operation—until the fetus has been dead for a month or longer. Our experience leads us to believe that the danger incident to the policy of expectancy is so great that if the fetus is dead, be that death recent or of some standing, it should be removed without delay. Exceptionally, our incision may carry us to the fetal sac. This occurs in some extra-peritoneal or broad ligament pregnancies, and the peritoneal cavity is not opened. In this variety the sac and placenta are entirely beneath the peritoneum. The latter may have been pushed up, stripped, as it were, from the anterior abdominal wall for a greater or less distance. Without disturbing the placenta, after having ligated the umbilical cord near the placenta, hastily remove the fetus. Evacuate the sac contents and then after separating the sac and the placenta from the surrounding structures to which they have become adherent, remove them. Usually our incision carries us into the peritoneal cavity. After placing the patient in the Trendelenburg position, separate the sac from the contiguous viscus or viscera to which it adheres. Control hemorrhage as you proceed. The general peritoneal cavity is protected by gauze compresses, which are numbered and counted, and then the incision is carried into the ovum. Occasionally you may be able to remove the ovum as a whole. If the placenta is not safely removable, if the nature of the adhesions of the surrounding organs to the ovum is such that their separation will prove disastrous, content yourself with evacuating the fetal cyst and then suturing its walls to the abdominal wound. The sac must be packed daily until the placenta has been expelled and the sac cavity obliterated. If the placenta is to be left behind, it is better that it be not disturbed.

The following methods have been employed:

1. The fetus, the umbilical cord, and the amniotic fluid have been removed. Everything else has been left in situ and the abdominal wall closed. This is an extremely risky experiment.

2. The fetus is removed and more or less of the sac is resected. Drainage of the sac cavity is employed, and the placenta and sac are left for spontaneous expulsion. This is the most frequently employed procedure.

3. After the removal of the fetus, umbilical cord and amniotic fluid, the placenta is removed in part—so much of it as is easily separated—and the remainder is left to spontaneous absorption.

4. The placenta is left in situ after removing the fetus. Then, after the expiration of a certain time, when it is hoped that the blood supply is spontaneously cut off, the placenta is shelled out.

5. The placenta and entire ovum are removed immediately. Ideal measure, if feasible.

6. The placenta and gestation sac are removed at once, likewise the neighboring organs, the ureters and ovaries, providing the hemorrhage cannot be arrested.

7. Preliminary ligature of the uterine and ovarian arteries of the side from which the placenta received its blood supply, followed by the removal of the placenta.

8. In some cases, the hemorrhage from the placental side was best controlled by inverting that part of the intestinal wall to which the placenta was attached. In other cases, the operators found it necessary to do a supra-vaginal hysterectomy and unilateral or bilateral ablation of the uterine appendages.

There is no disputing the fact that the fetal sac and placenta should be removed completely if the procedure be consistent with the safety of the mother. The complete ablation of the ovum is theoretically the only perfect operation.

The method we employ in those cases in which we fear to disturb the placenta is the following:

After incising the sac, removing the fetus and other intraovulary contents. and ligating the umbilical cord close to its implantation, we resect a portion of the sac wall and sew what is left to the abdominal wound, thus closing off the general peritoneal cavity. This leaves a large pouch, which is-packed with strips of aseptic gauze. Endeavor to keep this sac cavity aseptic until all placenta has sloughed out of the wound. The elimination of the placenta by this method takes from twenty to fifty days.

In some cases a vaginal drain has to be used, in addition to the abdominal drains. The first strips of gauze that are inserted in the fetal sac are made to serve the offices of a compress and a tampon. They are used to check the bleeding. After the first dressings, the gauze strips are used more with drainage in view. After the fetal cyst has been sewed to the abdominal wall, or immediately previous, according to exigencies of the case, the compresses that have been used to protect the general peritoneal cavity are removed. Sewing of the sac wall to the abdominal wound shuts off all communication between the cyst and the peritoneal cavity. We use No. 3 catgut to suture the sac wall to the abdominal wall. The abdominal wall is closed as in those cases in which a Mikulicz drain is employed. Post-operative treatment, symptomatic.

---

Insist on sanitary cigar factories and then use a public cigar cutter.

